# Surgery outside the Hospitals

**Published:** 1900-03-03

**Authors:** 


### Surgery Outside the Hospitals.

The general tendency of operative work to drift into the hands of hospital surgeons, which has made itself so manifest of recent years, is a matter of considerable interest to the medical profession. But it is really a matter of even more importance to mankind at large, for it would indeed be a sorry outcome of all the increase of knowledge which has been gained by so much labour if, instead of leading to greater perfection of surgical work among the mass of the profession, it were merely to lead to the concentration of surgical practice upon that small number within the profession who are able to obtain hospital appointments. Yet such has been the tendency. To many an aspiring surgeon a visit to a thoroughly up-to date operating theatre is a depressing experience, for he feels, and sometimes he is even made to feel, that to attempt to enter upon the higher walks of surgery without the aid of such helps and appliances as he finds there on every side, is almost an impertinence. And there is something to be said for such a view. The surgery of to-day does not concern itself merely with doing operations which are urgently necessary, and without the doing of which the patients would quickly die. Modern surgery is to a large extent a surgery of election. Operations are done not merely because they are necessary but because they are considered safe; this safety hinges almost absolutely upon the maintenance of asepsis; and if it is true that such a degree of asepsis as is essential for the safe performance of operative work can only be secured by the elaborate and costly surroundings provided in modern hospitals, then good-bye to surgery for the ordinary practitioner, and good-bye to the benefits of surgery for that large portion of mankind which dwells outside the reach of hospital treatment. Yet the tendency to restrict operative work to hospital surgeons is one which is strongly supported not only by the public, which always votes for specialism, but even among our profession. It is only quite recently that it was urged by a well-known American surgeon that "modern surgery is best done in hospitals, which can afford every facility for clinical investigation," the view taken being that the constant bacteriological testing of surgical technique, which is only possible where there is a perfectly equipped clinical laboratory, "is the first requisite for work of a high standard."

Although this is in a sense true, and although it is to be admitted that operative surgery of the highest type is most easily performed where every possible appliance is at hand, the teaching that the practice of surgery should be more and more confined to those who have these advantages, and that the rest of the profession should accept a humbler rôle, and should, in commercial phraseology, become the country agents of great operating firms, is one that we should very sorrowfully and reluctantly accept; and all the more so since recent surgical developments have shown that what may be called emergency surgery demands surgical skill of the highest type. If it were true that emergency surgery need only be a sort of "first aid" it might be reasonable to argue that the greatest good to the greatest number would be attained by ordinary surgeons restricting their ambitions to simple proceedings, and sending straight to hospital every complicated case. But this is no longer the position of affairs. Nothing is more clear than that if the best is to be done in purely emergency work the surgeon must be possessed of a high degree of surgical skill, and must be able to carry out his proceedings without delay and with the same degree of asepsis as is found necessary for the highest class of work done in hospitals. A man gored by a bull, a "run over" case with perhaps a rupture of the liver, a woman with a perforated ulcer of the stomach—all of them emergencies which may occur in the remotest country village—require for their proper treatment the highest type of operative surgery, and men able to tackle such emergencies properly need not stand aside from any surgery that is likely to come in their way.

Those then who despair of surgery outside hospitals are placed in a serious dilemma. Either emergencies must be bungled or surgery must be rendered possible even without the gorgeous appliances which we see in hospitals. Under these circumstances we turn with some relief to an address recently delivered by Mr. Lockwood, before the Leamington Medical Society, on the organisation of aseptic operations, in which he laid it down that the necessary perfection of asepsis can be attained by attention to details which can be carried out as satisfactorily, although perhaps not with the same ease, in a private house as in a public hospital. Everyone knows how high is the ideal of asepticity which Mr. Lockwood sets up, and how he has worked for years proving each step by which he hopes this ideal may be reached, and with this in view it is interesting to find him speaking as follows: "To my mind, one of the leading characteristics of aseptic surgery is its extreme simplicity. I do not know of any detail which cannot be carried out in a private house as well as in the most elaborate and expensive operation theatre." To the few the hospitals will perhaps always offer the highest skill and the greatest facilities. But in proportion as hospital machinery is glorified, and the popular superstition is spread abroad that without hospitals good surgery is impossible, the development of surgical skill among the profession at large will be held in check, much to the loss of mankind. Bacteriological investigation in clinical laboratories does good and admirable work in elevating the standard of asepsis within the hospitals. But for the benefit of the ordinary workaday world it perhaps does even better work still when it is able to evolve an organisation of operative proceedings, a routine if one likes to call it so, by the adoption of which a surgeon may cut himself adrift from his base, and without the support of a bacteriological laboratory, or the aid of an elaborately fitted operating theatre, may go out into the world and do good surgical work.

